# Comparing malaria risk exposure in rural Cambodia population using GPS tracking and questionnaires

**DOI:** 10.1186/s12936-024-04890-6

**Published:** 2024-03-12

**Authors:** Anaïs Pepey, Marc Souris, Saorin Kim, Thomas Obadia, Sophy Chy, Malen Ea, Sivkeng Ouk, Franck Remoue, Siv Sovannaroth, Ivo Mueller, Benoit Witkowski, Amélie Vantaux

**Affiliations:** 1 Malaria Molecular Epidemiology Unit, Institut Pasteur du Cambodge, 5 Blvd Monivong, Phnom Penh 120 210, Phnom Penh, BP983 Cambodia; 2 UMR Unité des Virus Emergents, UVE: Aix-Marseille Univ–IRD 190–Inserm 1207–IHU 5 Méditerranée Infection, 13005 Marseille, France; 3 Institut Pasteur, G5 Infectious Disease Epidemiology and Analytics, Université Paris Cité, 75015 Paris, France; 4 Institut Pasteur, Bioinformatics and Biostatistics Hub, Université Paris Cité, 75015 Paris, France; 5 UMR MIVEGEC, IRD, CNRS, University of Montpellier, Montpellier, France; 6 National Centre for Parasitology Entomology and Malaria Control (CNM), Phnom Penh 120 801, Phnom Penh, Cambodia; 7 The Walter and Eliza Hall Institute of Medical Research, Parkville, VIC Australia; 8 Department of Medical Biology, University of Melbourne, Melbourne, VIC Australia; 9 Present Address: Genetic and Biology of Plasmodium Unit, Institut Pasteur de Madagascar, Antananarivo, Madagascar

**Keywords:** Cambodia, GPS data loggers, Mobility, Anti-*Anopheles* saliva antibodies, Land use, Malaria, Transmission

## Abstract

**Background:**

The Great Mekong Subregion has attained a major decline in malaria cases and fatalities over the last years, but residual transmission hotspots remain, supposedly fueled by forest workers and migrant populations. This study aimed to: (i) characterize the fine-scale mobility of forest-goers and understand links between their daily movement patterns and malaria transmission, using parasites detection via real time polymerase chain reaction (RT PCR) and the individual exposure to *Anopheles* bites by quantification of anti-*Anopheles* saliva antibodies via enzyme-linked immunosorbent assay; (ii) assess the concordance of questionnaires and Global Positioning System (GPS) data loggers for measuring mobility.

**Methods:**

Two 28 day follow-ups during dry and rainy seasons, including a GPS tracking, questionnaires and health examinations, were performed on male forest goers representing the population at highest risk of infection. Their time spent in different land use categories and demographic data were analyzed in order to understand the risk factors driving malaria in the study area.

**Results:**

Malaria risk varied with village forest cover and at a resolution of only a few kilometers: participants from villages outside the forest had the highest malaria prevalence compared to participants from forest fringe’s villages. The time spent in a specific environment did not modulate the risk of malaria, in particular the time spent in forest was not associated with a higher probability to detect malaria among forest-goers. The levels of antibody response to *Anopheles* salivary peptide among participants were significantly higher during the rainy season, in accordance with *Anopheles* mosquito density variation, but was not affected by sociodemographic and mobility factors. The agreement between GPS and self-reported data was only 61.9% in reporting each kind of visited environment.

**Conclusions:**

In a context of residual malaria transmission which was mainly depicted by *P. vivax* asymptomatic infections, the implementation of questionnaires, GPS data-loggers and quantification of anti-saliva *Anopheles* antibodies on the high-risk group were not powerful enough to detect malaria risk factors associated with different mobility behaviours or time spent in various environments. The joint implementation of GPS trackers and questionnaires allowed to highlight the limitations of both methodologies and the benefits of using them together. New detection and follow-up strategies are still called for.

**Supplementary Information:**

The online version contains supplementary material available at 10.1186/s12936-024-04890-6.

## Background

Despite an international effort towards elimination, malaria persists with an estimated 247 million cases in 2021 [1]. In 2014, the Greater Mekong Subregion (GMS) committed to ending malaria transmission by 2030 and, since then, has witnessed considerable progress [2]. However, although *Plasmodium falciparum* incidence has drastically diminished, *Plasmodium vivax* burden remains high and challenge elimination [2] owing to its dormant liver-stages hypnozoites, involved in relapses [3], as well as its ability to circulate unnoticed and untreated thus leading to asymptomatic cases fuelling transmission [4].

In 2019, 85% of the estimated 32,197 malaria cases in Cambodia were represented by *P. vivax* [1, 2]. Mondulkiri province held the highest recorded malaria incidence in the GMS, with more than 50 cases per 1000 inhabitants from January to July 2019 [2]. Forest-related activities have been repeatedly identified as a major risk factor as the main malaria vectors (*Anopheles dirus* and *Anopheles minimus*) are forest-associated. Vector control tools to accelerate malaria elimination such as insecticide impregnated clothing or spatial repellents are yet to prove effective in the subregion [5]. Larviciding efficacy which requires landscape analyses to determine mosquito risk and its association to water bodies’ presence is limited by the large diversity of larval habitats type and size [6, 7].

Therefore and beside passive case detection (PCD), tools to accelerate malaria elimination are limited to active case detection (ACD), such as mass screening and treatment (MSAT) with the whole population targeted or focal screening and treatment (FSAT) restricted to high-risk groups, usually forest goers [8]. Reactive case detection has also been tested in different provinces but the very low detection rates and diagnostic sensitivity for asymptomatic participants did not demonstrate sufficient efficacy to be pursued [9, 10]. In Cambodia, ACD and PCD rely on immunochromatography-based rapid diagnostic tests (RDT) whose detection threshold has been documented at about 100 parasites/µL [11], making this method not sufficiently sensitive to detect most asymptomatic infections [5] with an elevated rates of false negatives [5, 12].

Modelling studies suggested that mass drug administration (MDA) to entire communities or sub-populations at the highest risk would be effective, but could facilitate the emergence of multi-drug resistant *P. falciparum* parasites [8, 13]. Programmatic implementation will likely also face difficulties to reach populations of interest at high coverage in remote areas, like deep forest [14, 15]. Recent findings suggest chemoprophylaxis would efficiently decrease malaria incidence, if implemented within the appropriate risk group [16].

An intensification of the elimination programme between 2018 and 2020 greatly decreased malaria cases nationwide, but infections from *P. vivax* were less affected than *P. falciparum* and mixed infections [17]. Primaquine is used as both a single dose treatment for *P. falciparum* since 2018 and a 14-day radical cure for *P. vivax* and *Plasmodium ovale* hypnozoites under certain conditions (such as e.g. prior glucose-6-phosphate dehydrogenase G6PD testing) since 2020 [18]. This treatment requires strict adherence to remain efficient and includes an important risk as it can trigger dose-dependent haemolysis in G6PD deficient patients, an X-linked genetic disorder found in 2 to 16% of the GMS population [19]. Screening for G6PD deficiency prior to primaquine administration is crucial for patient safety but difficult to implement [18]. Newly commercialized point of care tests have recently permitted quick and relatively easy G6PD deficiency screening, but they remained ambiguous for women as heterozygote individuals can give intermediate results difficult to interpret, and could not be implemented outside health centers [20]. Still, promising results indicate that G6PD testing might be implemented reliably outside these health centers soon, which would accelerate elimination [21].

The risk of false negatives, resistance emergence, insufficient coverage or haemolysis could be reduced by only prescribing anti-malarial drugs after identifying and diagnosing efficiently whom to treat. To target the adequate risk population, a population-oriented study could determine individual exposure and characterize the vector-host contact in transmission areas and ultimately paves the way for targeted intervention measures.

Although epidemiological questionnaires help characterize risk factors, other tools such as serology or GPS tracking are being used in heath studies to circumvent some of their limitations. Notably, because *P. vivax* induces relapses, an individual can suffer a malaria episode arising from an infectious bite that occurred weeks to months before [3]. For such cases, the use of immunological markers can provide insights about recent *Anopheles* bites. Indeed, *Anopheles* saliva compounds are secreted during the bite at the human skin level and could induce the production of specific anti-saliva immunoglobulins G (IgG) by bitten individuals that can be detected from blood samples [22]. It has been demonstrated in several studies that the intensity of the specific IgG antibody response to only one *Anopheles* salivary peptide (the gSG6-P1 peptide) is proportional to the level of exposure to bites received by the exposed individuals [23]. Hence, the IgG level to this salivary peptide has been validated as an adequate biomarker to measure the individual exposure to *Anopheles* vectors [22, 24].

Malaria transmission occurs at fine spatial scales [25, 26], and daily mobility patterns around areas with different malaria epidemiology settings can lead to imported cases and an increase of heterogeneity in individual disease risk [27, 28]. Fine-scale daily movement trajectories and knowledge of locations with increased *Anopheles* activity can inform public health intervention programs to progress towards elimination [29]. The standard methodology for measuring risk populations mobility in Cambodia relied on administering individual-based questionnaires [15, 30–32], which are likely to generate bias: a study from 2017 showed that participants were reluctant to admit to some of their travels, for example to illegal wood logging locations [33]. Here are reported the simultaneous collection of GPS tracks and standard self-reported questionnaires for a population at high risk of malaria. Individual fine-scale mobility patterns and their agreement with questionnaire data were measured, and the limitations arising from behavioral, technical and analytical aspects for both methods were explored [34]. Finally, to help refine targeted control strategies in the local context of *P. vivax* elimination, the interest of such fine-scale mobility metrics were evaluated.

## Methods

### Study site and population sample

**Fig. 1:**
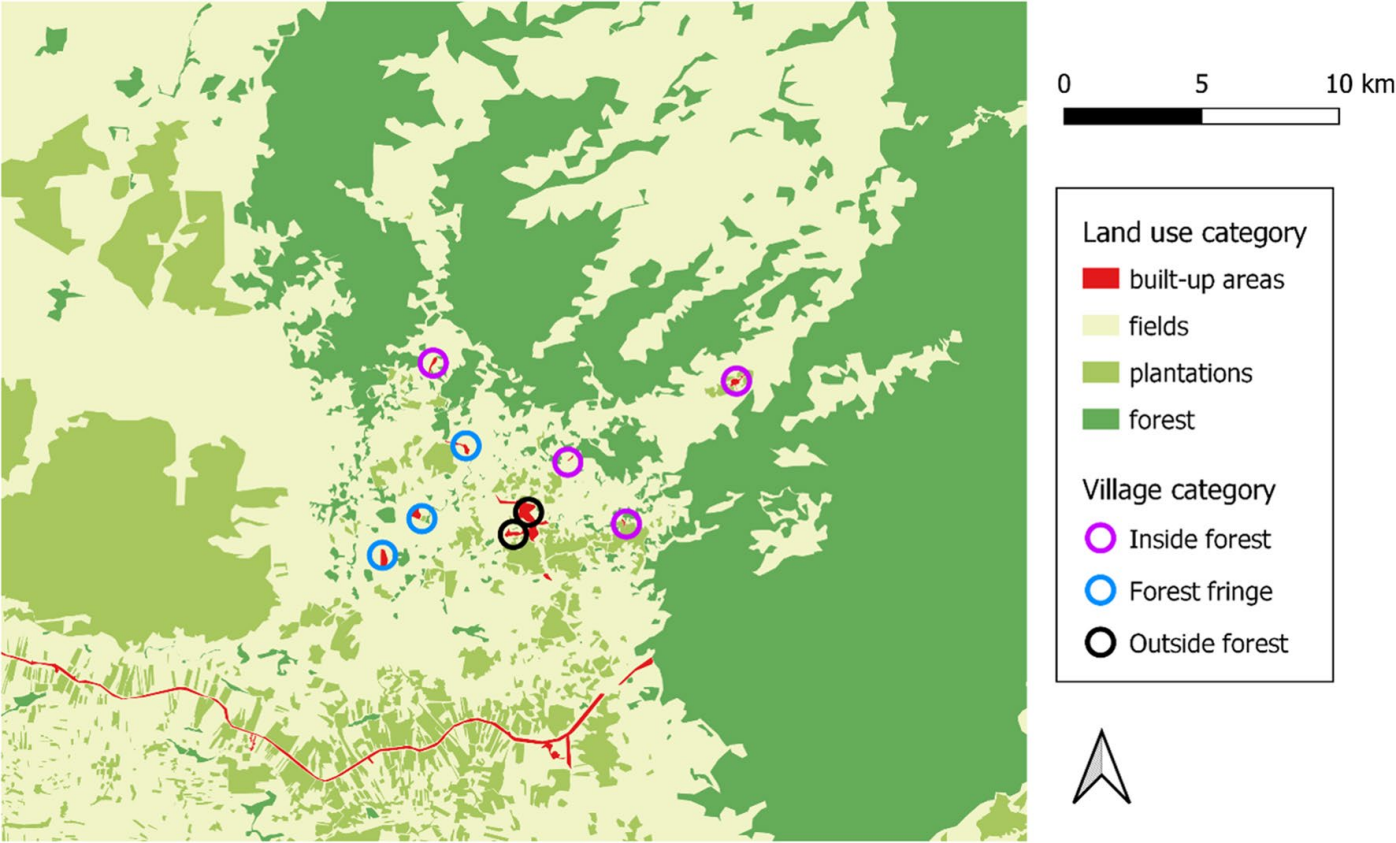
Land use and villages of the study area

The study site is located in Kaev Seima district, Mondulkiri province, North-Eastern Cambodia. Year is divided into rainy (May–October) and dry (November–April) seasons. Kaev Seima district is a rural, low-income environment where populations mainly live from agriculture and forest products exploitation. The land use of the study site was determined previously from high resolution satellite imagery (SPOT 6/7) captured in March 2018 (Fig. 1) [35]. The classification categorized the land into built-up areas (villages and roads), fields (incl. rice paddies, cassava culture), plantations (incl. banana, cashew nut, rubber trees) and forest. The nine villages were allocated to three categories according to their forest coverage around the households, following a previously defined gradient from inside to outside the forest [32].

The population sample included villagers at highest risk of malaria *i.e.* men between 13 and 60 years old [12, 30, 32]. To capture data from the two climate seasons, the study was designed with two rounds of collection, during the rainy (April to September 2018) and dry (February to April 2019) seasons. Participants were contacted and enrolled based on data collected in a prior cross-sectional study in the same district in 2017 [32], if they were available and willing to participate in the study, with half of them from malaria-negative and the other half from malaria-positive participants at the time of the cross-sectional study. A total of 160 participants for rainy season and 200 participants for dry season agreed to participate.

### Study design

**Fig. 2:**
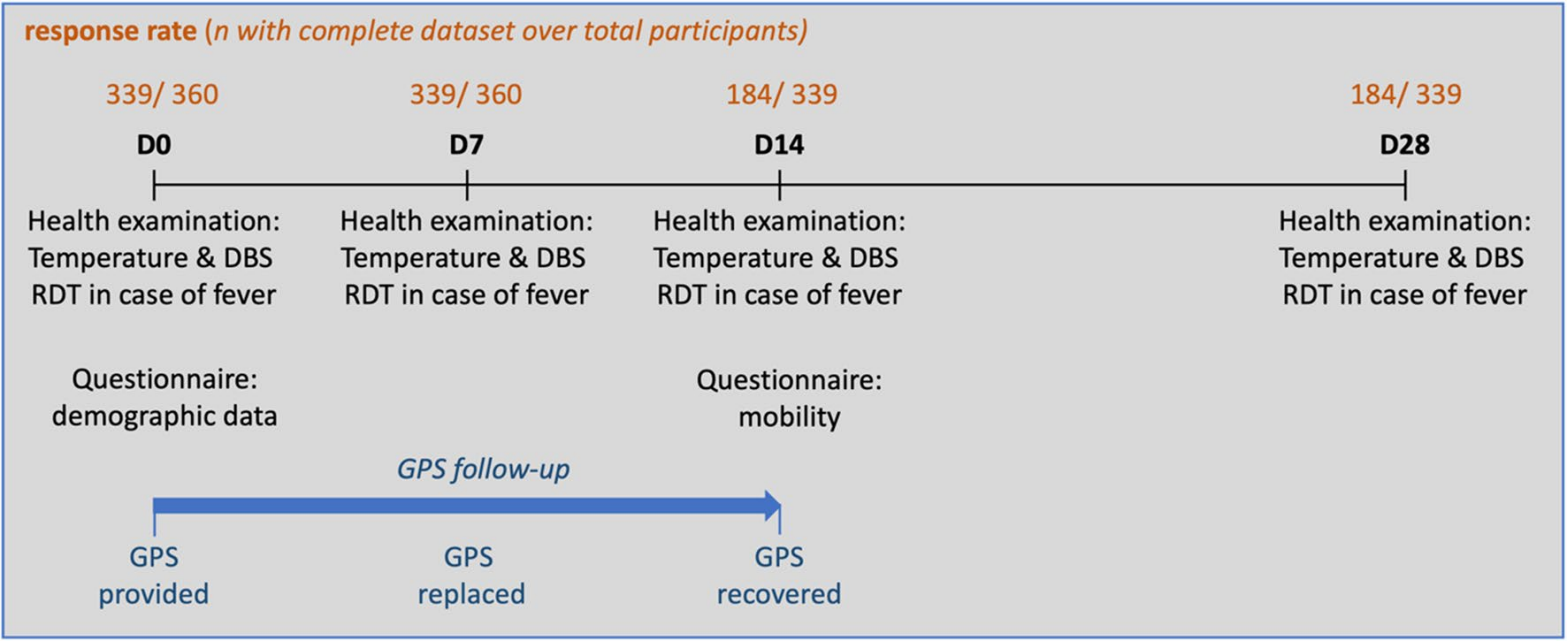
Study workflow including health examination, questionnaires, GPS data loggers distribution and collection, with the enrolment for each step

Household visits were scheduled on days 0, 7, 14 and 28 and included a short health examination, and questionnaires at day 0 (demographic data) and day 14 (mobility during the last 2 weeks, Fig. 2). The health examination on day 28 aimed at detecting *P. falciparum* infections which might have happened during the mobility study. A GPS data logger was provided from day 0 to day 14 with a planned exchange on day 7 for another fully charged device. The Igot-U GT600 GPS tracker (Mobile Action Technology Inc., Taipei, Taiwan) was selected to record participants’ mobility [36], as its battery life, data storage, price and unobtrusive design corresponded best to this study design and it had proven useful in other comparable studies [37, 38].

The questionnaire, designed in English and translated by a native Khmer speaker, gathered demographic data and information about recently visited environments. First round of self-reported data was collected on paper questionnaires and second round using the REDCap mobile app, an offline electronic data capture software linking to a REDCap server hosted at Institut Pasteur, Paris, France [39].

Health examination consisted of axillary temperature measure and collection of capillary blood onto filter paper. A RDT was performed in case of fever (temperature ≥ 37.5 °C, SD BIOLINE Malaria antigen Malaria Ag P.f./P.v). Participants testing positive were referred to a health centre to receive anti-malarial treatment.

### Laboratory procedures

DNA was extracted from filter paper blood spots with Instagene® Matrix (Bio-Rad, Courtaboeuf, France) according to the manufacturer’s instructions. Real-Time Polymerase Chain Reactions (RTPCR) were performed to detect malaria parasites and to carry out parasites speciation, as previously described [40]. RTPCR were carried out a posteriori and thus only symptomatic cases were referred to health centre. Briefly, a first RTPCR targeted the *Plasmodium* cytochrome b gene and determined the positive samples. Then, four nested RTPCR assays targeted the same gene specific to each species: *P. falciparum*, *Plasmodium malariae*, *P. ovale* and *P. vivax* [40], and a new protocol identified *Plasmodium knowlesi* with a new set of primers specific to the species (assay parameters and primers sequences are detailed in Additional file 1: Table S1).

The quantification of individual IgG responses to the specific *Anopheles* salivary peptide (gSG6-P1) was done by enzyme-linked immunosorbent assay (ELISA) on sera eluted from filter paper blood spot from the last day of follow-up (week 4) as previously described [23, 41] with some modifications (see Additional file material [Protocol S1]). The level of the specific IgG response was measured at the individual level and was expressed as the ΔOD, calculated as the difference between the mean of the individual optical densities (OD) in 2 antigen wells and the OD in 1 blank well containing no gSG6-P1 antigen. As a negative control, the specific anti-gSG6-P1 IgG response was also assayed in non-*Anopheles* exposed volunteers (N = 12) residing in an urban area with limited *Anopheles* mosquitoes' populations (Phnom Penh city) for more than three months: to quantify the nonspecific background antibody level and to calculate the cut-off value (calculated as the mean ΔOD_neg_ + 3 SDs) [23, 42, 43]. A participant was classified as an immune IgG responder if their ΔOD was > 0.539.

### Data processing and analysis

The GPS tracking data was curated and an optimal GPS dataset was produced consisting of participants willing to take the device daily and for whom trackers had logged enough time to be representative of individual trajectories [36]. The time spent in each land use category was extracted from participants GPS data along with descriptive metadata (day or night time and recorded speed) [36]. The time spent by a participant in an environment over the 2-week GPS follow-up period was computed both as the absolute “total hours” (cumulative duration logged by the device) and the relative “standardized time” (proportion of time logged relative to a 2 week period). Analyses were performed under two conditions: firstly, as night data (dropping “day” segments) when *Anopheles* vectors are most active and secondly as slow data (< 5 km/h, dropping higher velocity segments) when individuals are not driving a vehicle and can be exposed to mosquito bites, to determine which conditions led to increased malaria exposure for a participant.

A binomial variable representing malaria status was attributed to each participant: a value of 1 coded for at least one positive RTPCR for any species over the 4 week follow-up while 0 indicated participants with only negative samples. Sociodemographic variables were extracted from the questionnaire and coded as categorical: village of residence, village category (based on the forest cover), age category and main income.

The effects of season, age, main income, village category, village of residence, and time spent in different environments were tested on participants’ malaria status using univariate generalized linear mixed-effects models (GLMMs). To further understand participants’ mobility, univariate GLMMs were used to test the effect of travelled distance (standardized distance over 2 weeks: total, at night and at slow speed) on malaria status. These GLMMs were implemented with a binomial distribution. As participants visited different locations depending on the area they live in, the effect of the village on the time spent in each type of environment was also tested, using univariate GLMMs with a negative binomial distribution. Then, the village of residence was added as a covariate to control for a confounding effect in models testing the time spent in different environments on participants’ malaria status using univariate binomial GLMMs.

Anti-*Anopheles* saliva seropositivity was coded with a binomial variable depending on IgG titers and predetermined threshold. The effects of season, age, village category, village of residence, malaria status and time spent in different environments were tested on participants’ Anti-*Anopheles* saliva seropositivity (qualitative binomial model) and on their ΔOD value (quantitative Gaussian model, with log transformation of the variable to satisfy normality assumptions) using univariate GLMMs. The effect of the distance (standardized distance over 2 weeks: total, at night and at slow speed) on participants’ serological status was also tested using univariate GLMMs. Finally, the possible associations between the time spent in various environments and serological status, was tested while adjusting for village of residence as a possible confounder.

The questionnaire at day 14 enquired participants about any visit in the following environments during the two previous weeks: nearby forest, deep forest, cashew nut plantations, rubber plantations, rice fields and cassava fields. For each participant, visits to each land use category (extracted from their GPS tracks: at least 30 min logged at slow speed in one category) were compared to their declarations during the questionnaire at day 14, for each type of environment, using Pearson's Chi-squared tests. Each participant was attributed a binomial variable depending on this potential data discordance (1: discordance, 0: no discordance). GLMMs were used to assess the potential association between discordance and participants’ malaria status.

All the aforementioned GLMMs were implemented with participant ID coded as a random effect to account for repeated measurements from the same individual. Statistical significance was assessed with likelihood-ratio tests (LRT).

The groups of participants with fever, with a positive RTPCR result or with a symptomatic malaria infection from each season were compared using Pearson's Chi-squared tests.

Percentages are presented with exact confidence interval (CI), means with standard deviation (SD) and medians with interquartile range (IQR). All statistical analyses were conducted in R version 4.0 [44].

## Results

A total of 360 participants were invited to participate and enrolled but only 339 participants finally participated to the whole study.

## Study population

**Fig. 3:**
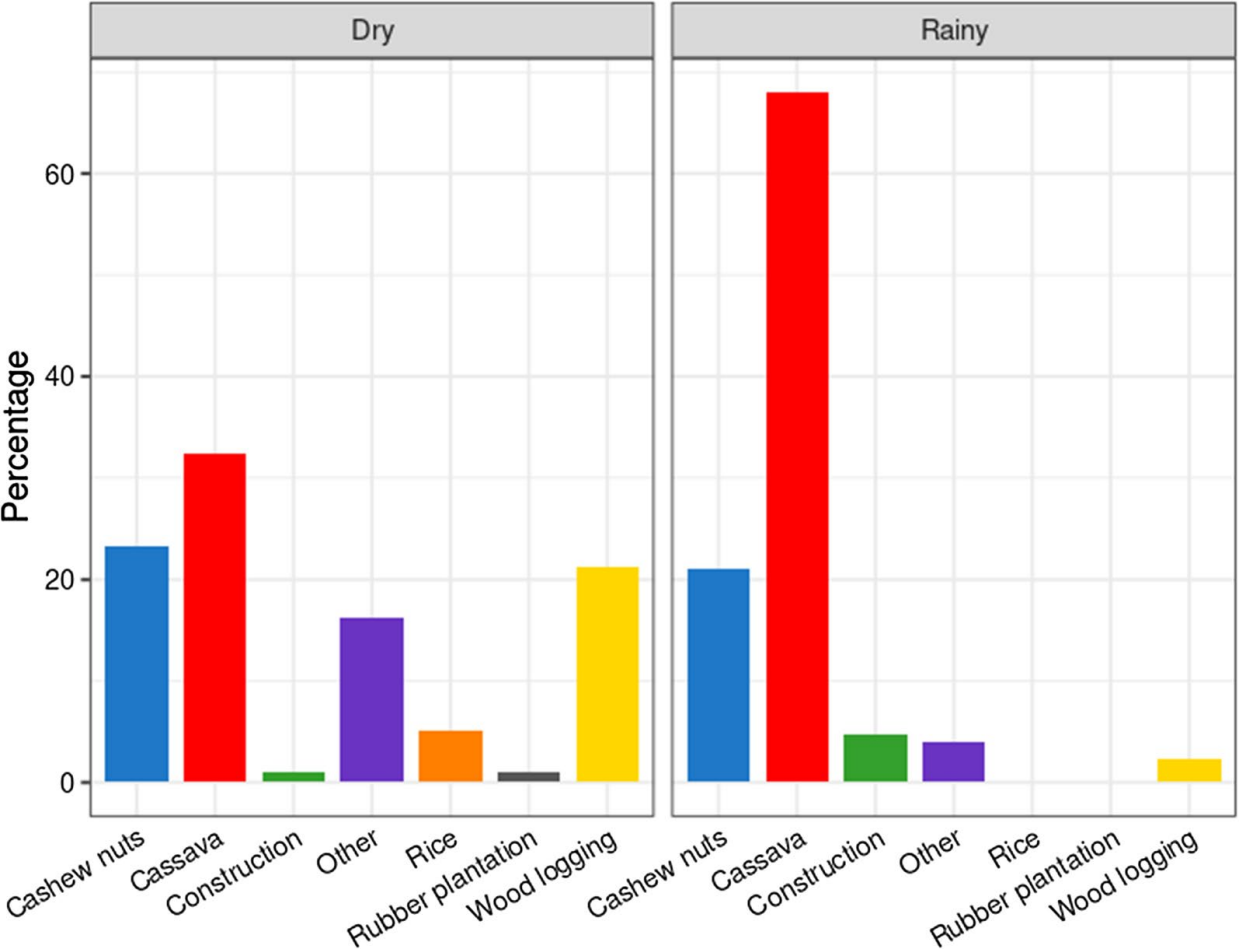
Main income declared by participants for each season during baseline questionnaire

Among the 339 respondents to the sociodemographic questionnaire, median age was 29 years old (IQR = 22, range 13–60, Additional file 1: Fig. S1). A majority of participants reported their main income source was related to agriculture (69.6%, CI [64.4–74.5%], Fig. 3).

At mid-follow-up on day 14, participants with a complete questionnaire in both seasons and PCR results (N = 184) reporting their recent travel history declared mostly short trips to the nearby forest (only one stay reported to last more than one day, Table 1). Visits to the deep forest corresponded to stays from less than a day to more than a week (over the last two weeks: 15.8% [10.8–21.8%] declared less than a day, 10.7% [6.8–16.3%] a day, 13% [8.5–18.8%] several days, 0.5% [0–3%] more than a week and 59.8% [52.3–67%] no visits). The preferred way of transportation was by motorbike, except for accessing the nearby forest and rice fields, for which walking was more frequent (Table S2). Although participants mainly declared to go to the deep forest with men from their family (37.8% [26.8–49.9%]) or co-workers (50% [38.1–61.9%]), they were also sometimes accompanied by women (8.1% [3.0–16.8%]) and children (4.1% [0.08–11.4%], Table S2). Women and children were often joining participants in cashew plantations (respectively 44.4% [34.9–54.3%] and 21.3% [14–30.2%]) and cassava fields (respectively 59.2% [44.2–73%] and 18.4% [8.8–32%], Table S2).

**Table 1:** Visits in the different environments during the past two weeks declared by the participants during the questionnaire at day 14, for all participants with a complete questionnaire at day 14 (N = 184)

Visits in the past 2 weeks	N	%	CI %
Nearby forest	141	76.6	[69.4–82.5]
Deep forest	74	40.2	[33.1–47.7%]
Rubber plantation	3	1.6	[0.3–4.7]
Cashew plantation	108	58.7	[51.2–65.9]
Cassava field	49	26.6	[20.4–33.6%]
Rice field	15	8.2	[4.6–13.1]

### Health examination

The epidemiology of malaria cases was highly consistent across collection rounds (Table 2). Malaria prevalence at baseline was high and similar between rainy and dry season (respectively 31.9% [24.7–39.7%] and 28.5% [22.3–35.3%], Chi-squared P = 0.56). Likewise, prevalence during follow-up remained stable (rainy: 50.6% [42.6–58.6%], dry: 43.5% [36.5–50.1%], Chi-squared P = 0.21). The proportion of symptomatic cases (rainy: 18.8% [10.1–30.5%], dry: 10.3% [4.8–18.7%]) did not significantly differ by season either (χ^2^_1_ = 1.53, P = 0.22). Notably, more participants experienced a fever during the rainy season compared to the dry season (22.7% [15.7–30.9%] vs. 12.5% [8.3–17.9%]; χ^2^_1_ = 5.14, P = 0.02) though it was not necessarily malaria-related (13.3% [7.9–20.4%] of participants with fever not due to malaria in rainy season and 8% [4.6–12.7%] in dry season).

**Table 2:** Malaria prevalence in rainy and dry seasons

Season	Rainy season						Dry season					
Week	Baseline			Over follow-up			Baseline			Over follow-up		
Value	N	%	CI %	N	%	CI %	N	%	CI %	N	%	CI %
*Plasmodium* spp.	51	31.9	[24.7–39.7]	81	50.6	[42.6–58.6]	57	28.5	[22.3–35.3]	87	43.5	[36.5–50.1]
*Plasmodium vivax*	44	27.5	[20.7–35.1]	64	40	[32.3–48]	47	23.5	[17.8–30]	78	39	[32.2–46.1]
*Plasmodium falciparum*	4	2.5	[0.7–6.3]	4	2.5	[0.7–6.3]	2	1	[0.1–3.6]	2	1	[0.1–3.6]
*P. vivax/ P. falciparum*	2	1.3	[0.2–4.4]	4	2.5	[0.7–6.3]	4	2	[0.5–5]	5	2.5	[0.8–5.7]
Undetermined	1	0.6	[0.01–3.4]	9	5.6	[2.6–10.4]	4	2	[0.5–5]	2	1	[0.1–3.6]

“Over follow-up” corresponds to individuals that have been positive at least once over the follow-up

*Plasmodium vivax* was the most frequent malaria parasite over both rainy and dry seasons, respectively found in 84% [74.1–91.2%] and 95.4% [88.6–98.7%] of detected infections. *Plasmodium falciparum* burden was 9.9% [4.4–18.5%] in rainy season and 8% [3.3–15.9%] in dry season among infections. Mixed infections were also uncommon accounting for 4.9% [1.4–12.2%] and 5.7% [1.9–12.9%] of cases. No carriage of *P. ovale, P. malariae* or *P. knowlesi* was detected, however a total of 16 positive samples could not be typed.

### GPS data

Battery life averaged 3 days and 7 h per device in the rainy season, and 4 days and 1 h in the dry season, with large heterogeneity between loggers (from a few minutes to 16 days and 18 h). The GPS devices logged on average 159.9 km by participant over the complete follow-up. Once the GPS tracks were curated, the average daily distance varied greatly among the 273 participants with optimal GPS tracks but was consistent between seasons and averaged 26 km per day per participant (median: 23.5 km, IQR: 14.4 km, range 10.2–97.3 km per day per participant, standardized distance over 2 weeks).

### Risk factors

Risk factors analysis was performed on a subset of participants having complete socioeconomic, malaria status and optimal GPS data (N = 258 participants, Fig. S2). Malaria status was not associated with the season, χ^2^_1_= 1.2, P = 0.27), the main income declared (χ^2^_8_  = 9.5, P = 0.3), nor with the village of residence χ^2^_8_= 14.1, P = 0.08). Malaria infections were more prevalent in adults between 21 and 39 years old (57.4% [47.5–66.9%]) than younger (42.5% [31.5–54.1%]) and older men (44.3% [32.4–56.7%]) over follow-up, but the difference was not significant (χ^2^_2_ = 5.3, P = 0.07). Malaria status was significantly affected by the village category (χ^2^_2_ = 7.4, P = 0.02): malaria prevalence in the villages on the forest fringe (36.4% [25.7–48.1]) was significantly lower than outside the forest (55.1% [46.4–63.7%]). Malaria prevalence for participants from villages inside the forest was not significantly different from the two other categories (53.3% [37.9–68.3%]; Additional file 1: Table S3).

Neither the total time nor the standardized time spent in a given land use were found to modulate the risk of malaria infection (Additional file 1: Table S4). Participants visited different parts of the study area depending on their village of residence (Additional file 1: Fig. S3) and there were significant differences between villages in the time spent in the forest at slow speed (χ^2^_8_ = 25.2, P = 0.001) and at night (χ^2^_8_ = 30.4, P < 0.001), and plantations at slow speed (χ^2^_8_ = 50.9, P < 0.001) and at night (χ^2^_8_ = 61.8, P < 0.001; Additional file 1: Table S5). However, there was still no effect of the time spent in a given land use on participants’ malaria status when the village of residence was added as a covariate to control for those village differences (Additional file 1: Table S6). Malaria status was not associated with the total distance travelled (malaria-positive: 27.4 km on average per day, IQR = 14.6 km vs. malaria negative: 25.3 km, IQR = 14.4 km, χ^2^_1_ = 2.20, P = 0.14), was not significant for the association to the distance travelled at night (malaria-positive: 6.5 km, IQR = 4.3 km, vs. malaria-negative: 5.6 km, IQR = 4.8 km; χ^2^_1_ = 3.79, P = 0.052) and was not associated to the distance travelled at slow speed (malaria-positive: 11 km, IQR = 8.4 km, vs. malaria-negative: 10.2 km, IQR = 7.3 km; χ^2^_1_ = 1.31, P = 0.25).

As high malaria prevalence at baseline was observed (30%), the analyses were also performed on a subset excluding the participants malaria-positive at D0 to determine the risk factors for incident infections only (N = 177), again no significant correlations were observed (Additional file 1: Tables S7, S8).

**Fig. 4:**
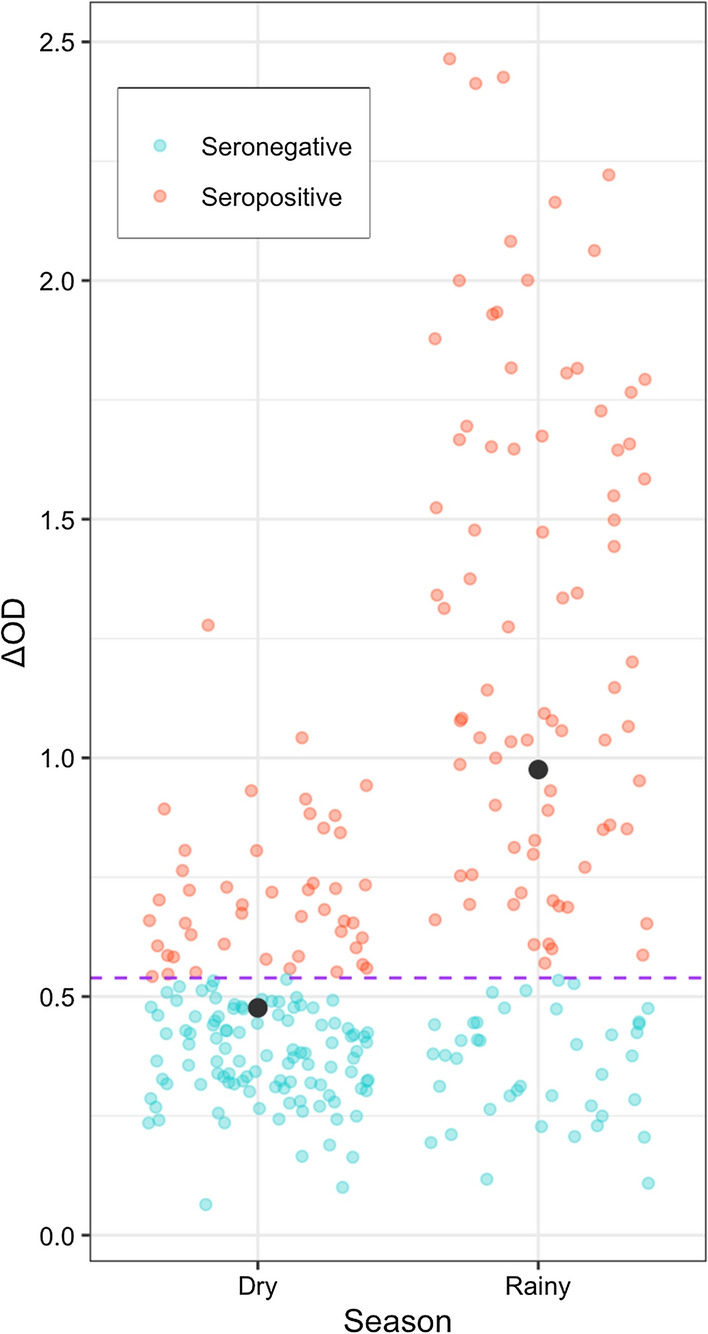
Participants’ serostatus and ΔOD by season (N = 270). The black dot indicates the mean ΔOD of each group (mean_dry_ = 0.476, mean_rainy_ = 0.976). The purple line indicates the cut-off value calculated form the negative controls (cut-off value = 0.539)

Immunological analyses were performed on a subset of participants that has complete malaria status, serology and GPS datasets (N = 270). In qualitative and quantitative univariate models respectively, seroprevalence and average ΔOD were significantly higher during rainy season compared to dry season (χ^2^_1_ = 35.36, P < 0.01 and χ^2^_1_ = 58.48, P < 0.01, respectively; Additional file 1: Table S9, Fig. 4). Participants from the villages on the forest fringe had the lowest average ΔOD (ΔOD = 0.62), followed by outside the forest (ΔOD = 0.69) and finally inside the forest (ΔOD = 0.87) though this did not reach statistical significance (χ^2^_1_ = 5.55, P = 0.06, Additional file 1: Table S9). All other factors such as malaria status, age and time spent in different environments had no significant association with seroprevalence and average ΔOD (Additional file 1: Table S9). The average ΔOD was significantly associated with the village of residence (χ^2^_8_ = 31.19, P < 0.001): the average ΔOD over all villages was 0.70, with 2 villages at the forest fringe and 2 inside the forest having the lowest average ΔOD (ranging 0.42–0.45) while Gaty, inside the forest, had the highest average ΔOD (1.09; Additional file 1: Table S10). When adding the village of residence as a covariate participants’ antibody levels were significantly positively associated with the total time spent in fields at night (χ^2^_1_ = 4.23, P = 0.04, Additional file 1: Fig. S4) and at slow speed (χ^2^_1_ = 4.03, P = 0.04, Additional file 1: Fig. S5) (Additional file 1: Table S11). No other effects were found (Additional file 1: Table S11). The analyses on seroprevalence with the time spent in different environments and village of residence as covariate did not converge due to small group’s size.

**Fig. 5:**
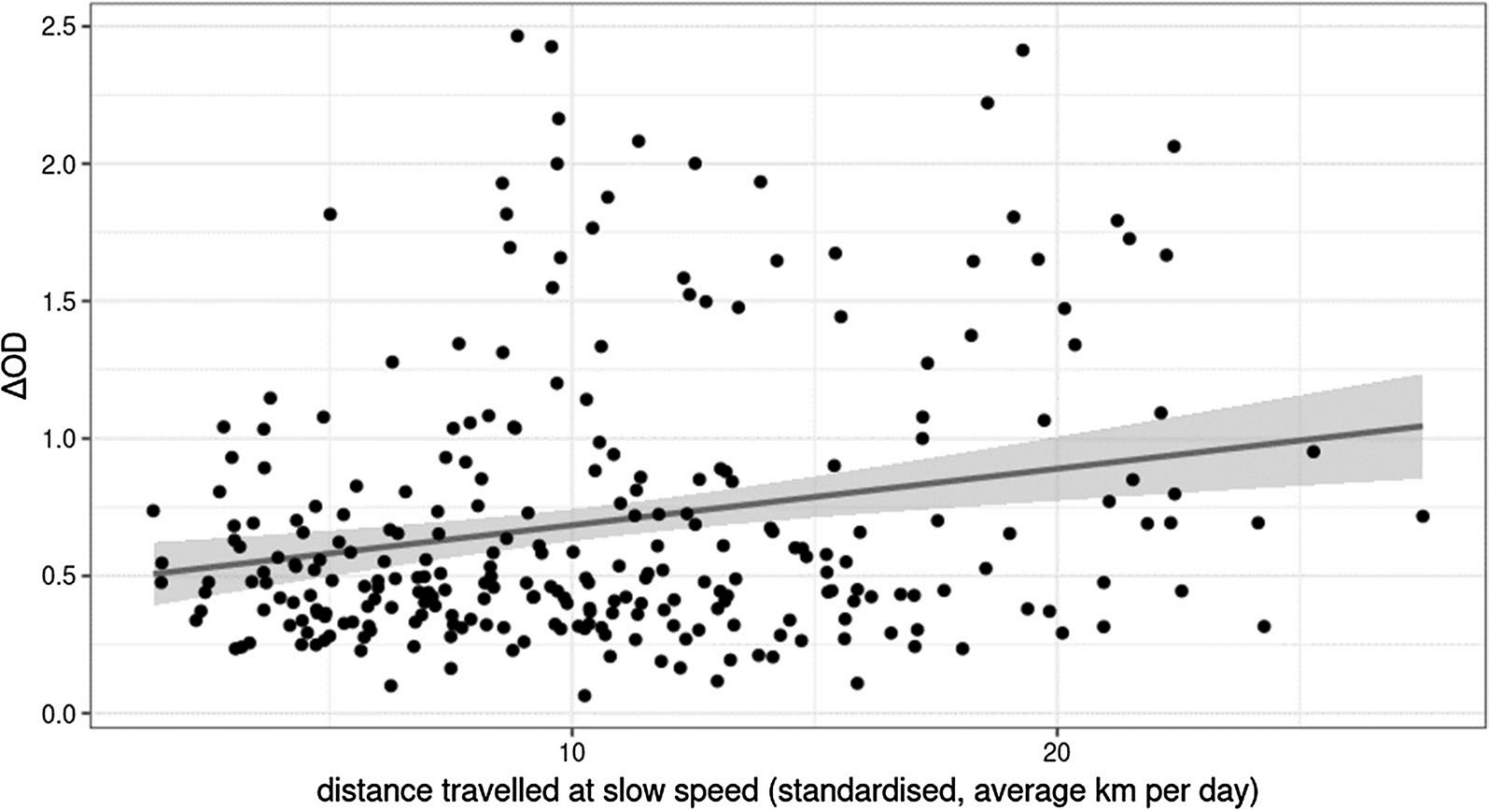
Association between average ΔOD and average standardized distance travelled at slow speed by day χ^2^_1_ = 10.2, P = 0.001, N = 270)

The total distance travelled during follow-up was not associated with seroprevalence (seropositive: 26.4 km on average per day, IQR = 14.3 km vs. seronegative: 25.5 km, IQR = 13.4 km, χ^2^_1_ = 0.38, P = 0.54) or with average ΔOD (χ^2^_1_ = 0.004, P = 0.95). Similarly, the overall distance travelled at night was not associated with seroprevalence (seropositive: 6.2 km on average per day, IQR = 3.6 km vs. seronegative: 5.8 km, IQR = 5.2 km, χ^2^_1_ = 0.63, P = 0.43) nor with the average ΔOD (χ^2^_1_ = 0.98, P = 0.32). However, the distance travelled at slow speed was positively associated with both seroprevalence (seropositive: 11.5 km on average per day, IQR = 8.2 km vs. seronegative: 9.8 km, IQR = 7.1 km, χ^2^_1_ = 0.7.1, P = 0.008) and average ΔOD (χ^2^_1_ = 10.2, P = 0.001, Fig. 5).

Overall, the most important findings were negative results: the time spent in a specific environment, even one identified at high risk of malaria such as the forest, was not correlated to a higher malaria prevalence. However, the time spent in fields under certain conditions did correlate with *Anopheles* bites exposure, in addition to the season and the total distance spent walking. Finally, the forest cover of the village of residence correlated both with malaria status and bites exposure (Table 3).

**Table 3:** Main findings

Risk factors	Prevalence %	Average per day	p-value
**Malaria status**			
Season			0.27
Dry	43.5		
Rainy	50.6		
Age category			0.07
Younger [≤ 20 years old]	42.5		
Mid [21–39 years old]	57.4		
Older [≥ 40 years old]	44.3		
Village of residence			0.08
Village category			**0.02**
Inside forest	53.3		
Forest fringe	36.4		
Outside forest	55.1		
Distance at slow speed			0.25
Positive		11 km	
Negative		10.2 km	
Time spent in any environment			> 0.05
**Serostatus**			
Season			** < 0.0001**
Dry	30.5		
Rainy	66.4		
Age category*			0.83–0.68
Younger [≤ 20 years old]	43.9		
Mid [21–39 years old]	48.2		
Older [≥ 40 years old]	45.9		
Village of residence			** < 0.001**
Gaty	60		
Ohchra	25		
Ohtrone	0		
Poucha	27.3		
Sraeampilkroam	16.7		
Sraeampilleu	65.7		
Sraektum	41.4		
Sraelvy	69.2		
Sraepreas	81.3		
Village category*			0.18–0.06
Inside forest	57.8		
Forest fringe	40.7		
Outside forest	45.8		
Distance at slow speed*			**0.008–0.001**
Seropositive		11.5 km	
Seronegative		9.8 km	
Time in fields at night**			**0.04**
Seropositive		17.8 h	
Seronegative		14.8 h	
Time in fields at slow speed**			**0.04**
Seropositive		18.8 h	
Seronegative		16 h	
Time spent in other environments			> 0.05

Significant differences (p-value < 0.05) are indicated in bold

One asterisk indicates p-values calculated respectively for qualitative and quantitative models. Two asterisks indicate p-values for quantitative models only with village of residence as covariate

### Comparison between questionnaire and GPS data

Compared to GPS, questionnaires significantly underestimated the visits in fields (48.7% (N = 96) declared a visit while 95.9% (N = 189) visits were observed in GPS data; χ^2^_1_ = 27.7, P < 0.0001) and overestimated the visits in the forest (91.4% (N = 180) for questionnaire versus 70.6% (N = 139) for GPS data; χ^2^_1_ = 109.7, P < 0.0001) and plantations (71.1% (N = 140) versus 61.4% (N = 121), χ^2^_1_ =, 4.1, P = 0.04).

For participants that had both a complete questionnaire dataset and optimal GPS (N = 197), each participant was assigned a variable corresponding to either concordant data (both GPS and questionnaire data demonstrated at least a visit or no visit at all) or not (only one dataset had evidence of a visit in the considered environment, Additional file 1: Table S12). The total concordance between the two datasets was 61.9%, with the highest value of concordance between visits in the forest (73.1%), followed by plantations (60.9%) and fields (51.8%). Concordance between the questionnaire and GPS datasets was not associated to malaria status over follow-up, over all visited environments (χ^2^_1_ = 1.03, P = 0.31), nor specifically between fields (χ^2^_1_ = 2.27, P = 0.13), plantations (χ^2^_1_ = 0.08, P = 0.78) or forest datasets (χ^2^_1_ = 0.57, P = 0.49).

## Discussion

The main objective of this study was to identify the factors associated with a gradation in infections at a fine scale to ultimately precise the transmission hotspots in Cambodia. As such, this study design aimed to determine whether the time spent in a certain land use category would correlate with malaria positivity, to ultimately enable identifying more accurately transmission areas. Notably, it was expected to observe that the participants spending more time in the forest, at peak biting hours and low speed, would be exposed to more infectious mosquito bites, resulting in an increased positivity rate. However, data from both GPS and questionnaire did not demonstrate any correlation. The continuous and swift decrease in *P. falciparum* prevalence, associated with increased *P. vivax* burden at the time of the study, which changed from 45% of cases being *P. vivax* or mixed infections in 2017 to 75% in 2018 [2], made it difficult to distinguish relapses from newly-infectious bites, the latter only being influenced by exposure to mosquitoes. Indeed, the aim was to associate malaria infection to the recent individual mobility data, which is not a direct link with *P. vivax* as the majority of infections are due to relapses that occur at a distant time from the initial infection [45, 46].

The questionnaire and GPS data were concordant for only 61.9% of the visits they included. Notably, the fields were overrepresented in the GPS data. That could by a consequence of the satellite imagery processing into a land use classification, which might categorize fallow lands as fields, or alternatively, because of outdated images or ongoing deforestation [47, 48], resulting in previously forest areas changing into fields. Such difference between the two datasets suggest that a complementary approach could be necessary to capture all aspects of malaria risk factors in Cambodia. Notably, illegal activities, often found associated with increased risk of malaria, would remain undetected by GPS-based collection methods.

The distance logged by the GPS devices at slow speed was not associated with malaria cases. Malaria risk was also not associated with a specific environment such as the forest. This result could highlight that malaria risk is important over all the study area, in all environments, or that malaria vectors are able to travel far from the forest or forest patches. Interestingly, the distance travelled at night time was not associated with malaria status, which could corroborate the importance of exposure at dusk, dawn, but also daytime [49].

Interestingly, the categorization of the villages according to the percentage of forest surrounding the households highlighted that the participants from villages outside the forest had a significantly higher malaria prevalence than those from villages at the forest fringes. Though sample size were unbalanced in that analysis (185 participants from outside the forest, 116 from forest fringes and 59 from inside the forest), they remained large enough to suggest an association between the village of residence and malaria exposure variations [32]. These results align with other studies highlighting the important exposure variability between villages only a few kilometers apart [12, 26]. Hypothetically, social or economic factors, such as family-privy gatherings or forest expeditions, particular agriculture methods, crops and forest patches affiliated to specific villages, and the size or the organization of a village, might have an impact on daily mobility, mosquito net usage and other risk behaviors, as previous results on human behavior variability [31, 50–52] and fine-scale exposure heterogeneity [12, 26] suggest. These factors could also explain how inhabitants from outside the forest display both the highest malaria and the second highest *Anopheles* bites exposure, while Cambodian primary vectors are forest-associated. In addition, the ELISA protocol cannot distinguish between exposure from primary vectors and other *Anopheles* species; the populations outside the forest could be exposed to important field or plantation-dwelling *Anopheles* species such as *Anopheles aconitus* or members of the *Anopheles hyrcanus* group [49, 53]. Finally, a recent mobility study from Laos using similar GPS loggers [54] did not analyse the association between malaria status and high-risk trips (dusk, dawn and night travels into the forest), but they could identify that socioeconomic factors increased the probability to engage in these trips: being 30 to 45 years old, having more than two children and sleeping outdoors without a structure.

The quantification of anti-*Anopheles* antibodies suggested that vector exposure was higher during the rainy season which is expected as *Anopheles* vector density increases in the rainy season, as it was previously observed in the GMS [6, 24, 55]. The levels of anti-*Anopheles* antibodies intensity response mirrored the trend of malaria prevalence. However, these analyses could not identify a significant association between anti-*Anopheles* antibodies levels and socioeconomic variables, malaria status or the time spent in a specific environment apart from the time spend in fields at night or slow speed when including the village of residence as a covariate. Particularly, there was no relation between the time spent at night or at slow speed in the forest, a land type generally associated with *Anopheles* bites exposure [15, 30, 31]. The lack of association between serological status and malaria status probably arises from the important incidence of *P. vivax* cases and their relapses, increasing the time spent between an infectious bite and a detected malaria case.

The three methodologies implemented during this study—questionnaires, GPS loggers and vector exposure serological marker—could not identify the mobility patterns and environments responsible for malaria cases in the study area. However, rather than the methods themselves, the context might not be suitable to their implementation. The ΔOD values followed the expected seasonal abundance of *Anopheles* and most of the GPS data was optimal (75% of the participants had optimal GPS data). Both methodologies were successfully implemented elsewhere to characterize vector exposure [22, 24] and mobility associated to malaria transmission [37, 56, 57]. However, malaria prevalence is sharply decreasing in Cambodia. *Plasmodium falciparum* cases represent only a fraction of infections, meaning that GPS trackers alone are not suitable to characterize the origin of most malaria infections. Infectious bites constitute only a very small percentage of mosquito bites, making vector exposure quantification less relevant as prevalence decreases. Finally, a different approach to mobility might be required to understand its association to malaria risk, in which mobility is not subpopulation-dependant but a process joining risk groups through migration and cases importation [58]. A study implemented at a bigger scale, looking at long-distance travels could help understand malaria transmission across villages, risk areas and demographic groups. Indeed, sample size was a limitation in this study, considering the important rate of unusable data (only 258 out of 341 participants with complete socioeconomic, malaria status and optimal GPS data) and the small effect size observed. A retrospective power and sample size calculations were perfomred using the package simr [59], based on 1000 simulations. This dataset was largely underpowered with a power of 13.9% for the analysis of time spent at night in the forest and 3.1% for the time spent at slow speed in the forest. Keeping the effect sizes observed in this study, a sample size of 500 and 1200 participants, for testing the hypothesis that malaria risk was correlated to the time spend at night or at low speed in the forest, respectively, would be needed to reach 80% power.

As such, both GPS tracking and vector exposure serological marker quantification do not appear to provide the resolution required to identify risk factors associated to malaria cases, mostly relapsing *P. vivax* infections, in the current Cambodian context. An FSAT approach targeting people with fever would have limited efficacy as only 12.5% (21 symptomatic cases for 168 positive RTPCRs) participants with an ongoing malaria infection had fever. Likewise, RDT-based malaria detection is also not suited to such low transmission settings, where the remaining hotspots often consist of sub-microscopic infections evading detection. Since many subclinical *P. vivax* cases remain undetected, testing for recent *P. vivax* infections via the use of serological exposure markers could be a more adapted elimination strategy [60, 61]. The modelling of *P. vivax* serological testing and treatment (*Pv*SeroTAT) demonstrated that it could significantly reduce *P. vivax* transmission while avoiding overtreatment, thus reducing the risk of accidental haemolysis in G6PD deficient patients [61]. The *Pv*SeroTAT strategy, targeting both blood and liver stages parasites, appears to be a safer and efficient alternative to MDA, while also more efficient than MSAT [61].

## Conclusion

In a context of residual malaria transmission which is mainly depicted by *P. vivax* asymptomatic infections, the implementation of questionnaires, GPS data-loggers and ELISA quantification of anti-*Anopheles* antibodies detection on the high-risk group were not specific enough to detect malaria risk factors associated with different mobility behaviors or time spent in various environments. The joint implementation of GPS trackers and questionnaires allowed to highlight the limitations of both methodologies and the benefits of using them together. The collected data characterized forest-goers mobility, the social context of their visits in different environments, in addition to quantitative data about the time and distance spent in these environments. New detection and follow-up strategies are still called for.

### Supplementary Information

**Additional file 1.** Additional tables and figures.

## Data Availability

The de-identified datasets generated and/or analysed during the current study is being made publicly available in a Zenodo repository. In the meantime, it is available from the corresponding author on reasonable request.

## Abbreviations

ACD: Active case detection
CI: Confidence interval
DBS: Dried blood spot
df: Degrees of freedom
ELISA: Enzyme-Linked Immunosorbent Assay
FSAT: Focal screening and treatment
IgG: Immunoglobulin G
IRS: Indoor residual spraying
GLMM: Generalized linear mixed models
GMS: Great Mekong Subregion
GPS: Global positioning system
LTR: Likelihood-ratio test
MSAT: Mass screening and treatment
OD: Optical density
OR: Odd ratio
PCD: Passive case detection
*Pv*SeroTAT: *Plasmodium vivax* Serological testing and treatment
RDT: Rapid diagnostic test
REDCap: Research electronic data capture
RTPCR: Real time polymerase chain reaction
ST: Standardized time
WHO: World Health Organization
