# Supplementary material for: Comparing malaria risk exposure in rural Cambodia population using GPS tracking and questionnaires
Source: Malar J. 2024 Mar 12;23:75. doi: 10.1186/s12936-024-04890-6 (PMC10936104; doi:10.1186/s12936-024-04890-6)
Supplement: Supplementary file 1 — Additional file 1. Additional tables and figures. [file 12936_2024_4890_MOESM1_ESM.docx]

Comparing malaria risk exposure in rural Cambodia population using GPS tracking and questionnaires

Anaïs Pepey^1^, Marc Souris^2^, Saorin Kim^1^, Thomas Obadia^3,4^, Sophy Chy^1^, Malen Ea^1^, Sivkeng Ouk^1^, Franck Remoue^5^, Siv Sovannaroth^6^, Ivo Mueller^7,8^, Benoit Witkowski^1*ψ^, Amélie Vantaux^1*ψ^

# Additional file 1: Additional tables and figures

Table S1: Primers sequences and real-time PCR conditions used to detect *Plasmodium* species.

*35 cycles when nested real-time PCR, 45 cycles when real-time PCR alone.

| PCR | **Primer** | **Sequence (5'-3')** | **Master Mix** | **Assay parameters** |
| --- | --- | --- | --- | --- |
| Real-time PCR screening | | | | |
| **Screening PCR** | RTPCRScreening2_F | TGGAGTGGATGGTGTTTTAGA | Hot FirePol EvaGreen qPCR Mix Solis Biodyne 1X (#08-24-00020), Primers 150 nM, 5 μl DNA template.  Total volume 20 μl | 95°C-15 min; 45 cycles: 95°C-15 sec/60°C-20 sec/72°C-20 sec 95°C-2 min 68°C-2 min |
|  | RTPCRScreening2_R | TTGCACCCCAATARCTCATTT |  |  |
| Nested real-time PCR species | | | | |
| **Primary PCR** | RTPCRScreening2_F | TGGAGTGGATGGTGTTTTAGA | Hot FirePol DNA Pol. Solis BioDyne 1.25 U (#01-02-01000), dNTP 200 μM, MgCl2 2.5mM, Primers 250 nM, 5 μl DNA template.  Total volume 20 μl | 94°C −15 min 20 cycles: 94°C-30 sec/ 58°C −1 min/ 72°C-1 min 72°C-10 min |
|  | RTPCRSreening3_R | ACCCTAAAGGATTTGTGCTACC |  |  |
| **Nested real-time PCR Pf** | Pf_RTPCR_F | ATGGATATCTGGATTGATTTTATT  TATGA | Hot FirePol EvaGreen HRM Mix Solis Biodyne 1X (#08-33-00001), Primers 250 nM, 5 μl template Primary PCR products 1:10.  Total volume 20 μl | 95°C-15 min 35–45 cycles*: 95°C-10 sec/62°C-20 sec/72°C-25 sec 95°C-1 min 40°C-1 min |
|  | Pf_RTPCR_R | TCCTCCACATATCCAAATTACTGC |  |  |
| **Nested real-time PCR Pv** | Pv_RTPCR_F | TGCTACAGGTGCATCTCTTGTATTC |  |  |
|  | Pv_RTPCR_R | ATTTGTCCCCAAGGTAAAACG |  |  |
| **Nested real-time PCR Pm** | Pm_RTPCR_F | ACAGGTGCATCACTTGTATTTTTTC |  |  |
|  | Pm_RTPCR_R | TGCTGGAATTGAAGATAATAAATTA  GTAATAACT |  |  |
| **Nested real-time PCR Po** | Po_RTPCR_F | GTTATATGGTTATGTGGAGGATATA  CTGTT |  |  |
|  | Po_RTPCR_R | CGAATGGAAGAATAAAATGTAGTA  CG |  |  |
| **Nested real-time PCR Pk** | Pk_RTPCR_F | CTCCAGAAATTTCTTACGCATACTAC |  |  |
|  | Pk_RTPCR_R | CCCAAGGTAAAACATAACCTATAA  AA |  |  |

Protocol S1: Anti-*Anopheles* saliva quantification by ELISA:

The wells were coated with 100 μL of gSG6-P1 peptide solution (20µg/mg) for 2h30min at 37^o^C. Plate wells were then blocked for 1h at 37^o^C using 200 μL of blocking buffer, then washed. Individual sera were incubated in 100 μL duplicate at 4^o^C overnight at a 1/10 dilution for DBS eluate and 1/40 for serum (in PBS—Tween 0.1%). After washing the plate, secondary human IgG in PBS-tween 1% (final concentration 1/1000) was added and incubated at 37^o^C for 1h30min, then was washed again. The signal peptide (final concentration 1/1000) was incubated at 37^o^C for 1h, then washed. Colorimetric development was carried out using ABTS in 0.05M citrate buffer (pH=4) containing 0.003% H_2_O_2_ and incubated at room temperature for 2h. Absorbance (OD) was measured at 415 nm.


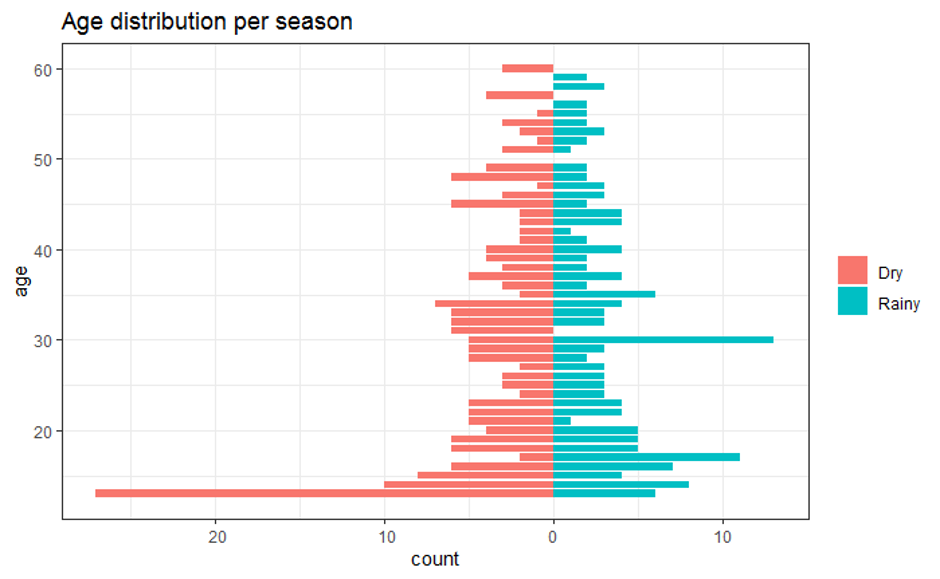


Figure S1: Age distribution among study population for dry and rainy season (N = 360).

Table S2: Transportation and relatives going to the different environments with the participants at least once. Each category respectively encompasses *i)* children: kids and grandkids; *ii)* women: all women from family but daughters (sisters, wives, mothers, etc.); *iii)* men: all men from the same family but sons (brothers, fathers, etc.); *iv)* co-workers: unrelated adult people working with them. Data from all participants declaring their visits at day 14 (N = 184).

| Visits | N | % | CI | Relatives | N | % | CI | Transportation | N | % | CI |
| --- | --- | --- | --- | --- | --- | --- | --- | --- | --- | --- | --- |
| Nearby forest | 141 | 76.6 | [69.8 - 82.5%] | Children | 5 | 3.5 | [1.2 - 8.1%] | Walking | 123 | 87.2 | [80,6 – 92.3%] |
|  |  |  |  | Women | 7 | 5 | [2.0 - 9,0%] | Bike | 17 | 12.1 | [7.2 - 18.6%] |
|  |  |  |  | Men | 9 | 6.4 | [3.0 - 11.8%] | Car | 0 | 0 | [0 – 2.6%] |
|  |  |  |  | Coworkers | 11 | 7.8 | [4.0 - 13.5%] |  |  |  |  |
| Deep forest | 74 | 40.2 | [33.1 - 47.7%] | Children | 3 | 4.1 | [0.08 - 11.4%] | Walking | 4 | 5.4 | [1.5 - 13.7%] |
|  |  |  |  | Women | 6 | 8.1 | [3.0 - 16.8%] | Bike | 65 | 87.8 | [78.2 - 94.3%] |
|  |  |  |  | Men | 28 | 37.8 | [26.8 - 49.9%] | Car | 4 | 5.4 | [1.5 - 13.3%] |
|  |  |  |  | Coworkers | 37 | 50 | [38.1 - 61.9%] |  |  |  |  |
| Rubber plantation | 3 | 1.6 | [0.3 - 4.7%] | Children | 0 | 0 | [0 – 70.8%] | Walking | 1 | 33.3 | [0.8 - 90.6%] |
|  |  |  |  | Women | 0 | 0 | [0 – 70.8%] | Bike | 2 | 66.7 | [9.4 - 99.2%] |
|  |  |  |  | Men | 1 | 33.3 | [0.8 - 90.6%] | Car | 0 | 0 | [0 – 70.8%] |
|  |  |  |  | Coworkers | 0 | 0 | [0 – 70.8%]  0 |  |  |  |  |
| Cashew plantation | 108 | 58.7 | [51.2 - 65.9%] | Children | 23 | 21.3 | [14.0 - 30.2%] | Walking | 25 | 23.1 | [15.6 - 32.2%] |
|  |  |  |  | Women | 48 | 44.4 | [34.9 - 54.3%] | Bike | 79 | 73.1 | [63.8 - 81.2%] |
|  |  |  |  | Men | 19 | 17.6 | [10.9 - 26.1%] | Car | 1 | 0.9 | [0.02 - 5.0%] |
|  |  |  |  | Coworkers | 3 | 2.8 | [0.6 - 7.9%] |  |  |  |  |
| Cassava field | 49 | 26.6 | [20.4 - 33.6%] | Children | 9 | 18.4 | [8.8 - 32.0%] | Walking | 8 | 16.3 | [7.3 - 29.7%] |
|  |  |  |  | Women | 29 | 59.2 | [44.2 - 73.0%] | Bike | 41 | 83.7 | [70.3 - 92.7%] |
|  |  |  |  | Men | 14 | 28.6 | [16.6 - 43.3%] | Car | 0 | 0 | [0 – 7.3%] |
|  |  |  |  | Coworkers | 3 | 6.1 | [1.3 - 16.9%] |  |  |  |  |
| Rice field | 15 | 8.2 | [4.6 - 13.1%] | Children | 1 | 6.7 | [0.2 - 31.9%] | Walking | 9 | 60 | [32.3 - 83.7%] |
|  |  |  |  | Women | 2 | 13.3 | [1.7 - 40.5%] | Bike | 6 | 40 | [16.3 - 67.7%] |
|  |  |  |  | Men | 1 | 6.7 | [0.2 - 31.9%] | Car | 0 | 0 | [0 – 21.8%] |
|  |  |  |  | Coworkers | 1 | 6.7 | [0.2 - 31.9%] |  |  |  |  |


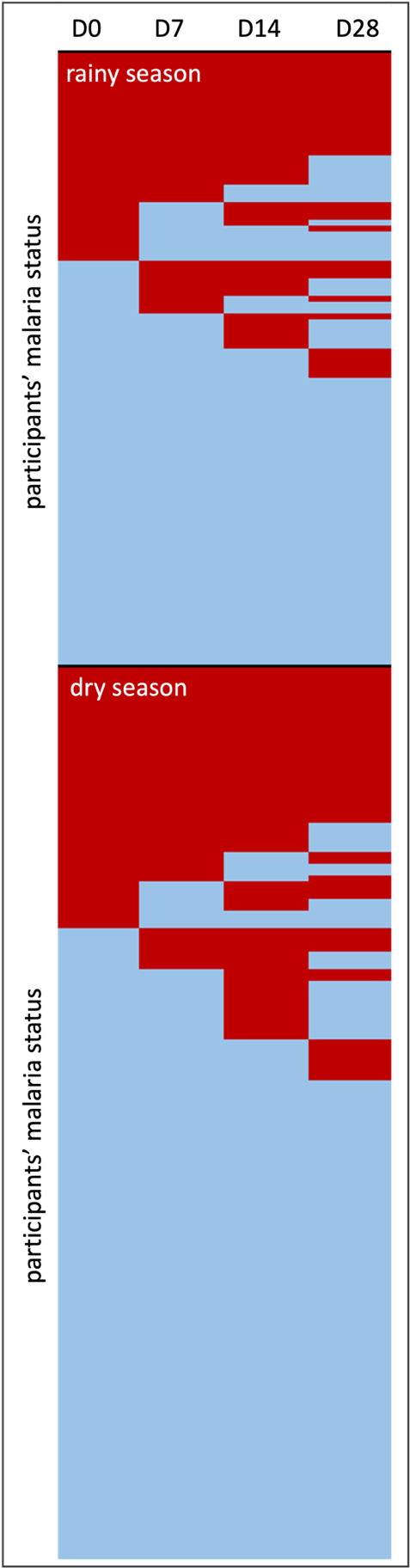


Figure S2: Participants’ incidence (red: RTPCR positive, blue: negative) at day 0, day 7, day 14 and day 28 of follow-up over rainy season (N = 105) and dry season (N = 153) from participants with a complete dataset (N = 258).


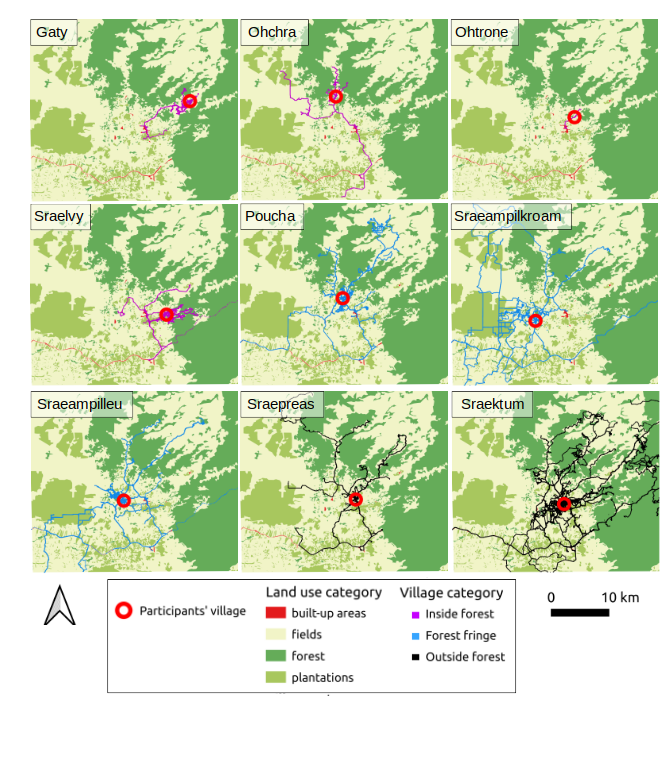


Figure S3: Optimal GPS points of participants by villages inside the forest [Gaty (N = 10), Ohchra (N = 8), Ohtrone (N= 1), Sraelvy (N = 26)], at the forest fringes [Poucha (N = 22), Sraeamplikroam (N = 27), Sreaempilleu (N = 28)] and outside the forest [Sreapreas (N = 119) and Sraektum (N = 17)].

Table S3: Tukey post-hoc test results on the prevalence over follow-up of each village age category. Significant results are in bold.

| **Pairwise comparison** | **OR** | **OR 95% CI** | **P-value** |
| --- | --- | --- | --- |
| Forest fringe - Inside forest | 2.17 | [1.32 – 3.02] | 0.17 |
| Inside forest - Outside forest | 1.08 | [0.33 – 1.83] | 0.98 |
| Forest fringe - Outside forest | 2.34 | [1.67 – 3] | **0.03** |

Table S4: GLMMs comparisons of malaria status and time spent in a category of land use on optimal data, using all data, night (18:00 to 5:59) data and slow (<5 km/h) data for all participants with a valid optimal GPS dataset (N = 258).

| **Dataset** | **Land use**  **category** | **Time**  **variable** | **χ^2^_1_** | **P-value** |
| --- | --- | --- | --- | --- |
| night | forest | Standardised time | 0.02 | 0.89 |
|  | fields |  | 0.04 | 0.84 |
|  | plantations |  | 0.37 | 0.54 |
|  | village |  | 0.07 | 0.79 |
|  | forest | Total hours | 1.74 | 0.19 |
|  | fields |  | 0.56 | 0.45 |
|  | plantations |  | 0.24 | 0.63 |
|  | village |  | 0.09 | 0.77 |
| slow | forest | Standardised time | 0.01 | 0.93 |
|  | fields |  | 1.02 | 0.31 |
|  | plantations |  | 0.04 | 0.85 |
|  | village |  | 0.39 | 0.53 |
|  | forest | Total hours | 0.34 | 0.56 |
|  | fields |  | 3.04 | 0.08 |
|  | plantations |  | 0.30 | 0.58 |
|  | village |  | 0.29 | 0.59 |

Table S5: Standardised time (in decimal hours) spent in average in forest and plantations over follow-up per village (N = 258).

| Village | Village category | N | Forest  slow speed | Forest  at night | Plantations  slow speed | Plantations  at night |
| --- | --- | --- | --- | --- | --- | --- |
| Gaty | Inside forest | 10 | 2.3 | 0.1 | 24.6 | 33.5 |
| Ohchra |  | 8 | 12 | 2.2 | 3.1 | 0.2 |
| Ohtrone |  | 1 | 0.6 | 2 | 0.2 | 0.1 |
| Sraelvy |  | 26 | 15.8 | 15.3 | 69.7 | 74.8 |
| Poucha | Forest fringe | 22 | 6.6 | 1.4 | 0.8 | 0.4 |
| Sraeampilkroam |  | 27 | 22.6 | 20.5 | 22.8 | 20.7 |
| Sraeampilleu |  | 28 | 54.4 | 51.4 | 32.1 | 31.6 |
| Sraektum | Outside forest | 119 | 28.2 | 19.3 | 29.8 | 31.9 |
| Sraepreas |  | 17 | 21.1 | 9.5 | 4.7 | 2.2 |
| *Total* | ***-*** | ***258*** | ***25.3*** | ***19*** | ***28.1*** | ***29.3*** |

Table S6: GLMMs comparisons of malaria status and time spent in a category of land use with village of residence as a covariate. Time variable was extracted from optimal data, as night data (18:00 to 5:59) or slow data (<5 km/h) for all participants with a valid optimal GPS dataset (N = 258).

| **Dataset** | **Land use**  **category** | **Time**  **variable** | **χ^2^_1_** | **P-value** |
| --- | --- | --- | --- | --- |
| night | forest | Standardised time | 0.22 | 0.64 |
|  | fields |  | 0.06 | 0.81 |
|  | plantations |  | 0.37 | 0.54 |
|  | village |  | 0.11 | 0.74 |
|  | forest | Total hours | 1.13 | 0.29 |
|  | fields |  | 0.09 | 0.77 |
|  | plantations |  | 0.34 | 0.56 |
|  | village |  | 0.11 | 0.74 |
| slow | forest | Standardised time | 0.10 | 0.75 |
|  | fields |  | 0.11 | 0.74 |
|  | plantations |  | 0.30 | 0.58 |
|  | village |  | 0.004 | 0.95 |
|  | forest | Total hours | 0.40 | 0.52 |
|  | fields |  | 0.20 | 0.65 |
|  | plantations |  | 0.22 | 0.64 |
|  | village |  | 0.13 | 0.71 |

Table S7: GLMMs comparisons of malaria status and season, socioeconomic variables and distance for all participants with incident infections and with a valid optimal GPS dataset (N = 177). The village GLMM induced a singular convergence due to small sample size and the results are not included.

| Variable | χ^2^_1_ | P-value |
| --- | --- | --- |
| season | 0.53 | 0.47 |
| **Socioeconomic factors** |  |  |
| main income | 8.48 | 0.29 |
| village category | 2.11 | 0.35 |
| age category | 2.44 | 0.30 |
| **Distance** |  |  |
| all | 0.02 | 0.88 |
| slow | 1.32 | 0.25 |
| night | 0.05 | 0.08 |

Table S8: GLMMs comparisons of malaria status and time spent in a category of land use. Time variable was extracted from optimal data, as night data (18:00 to 5:59) or slow data (<5 km/h) for all participants with incident infections and with a valid optimal GPS dataset (N = 177). Analysis with village of residence as a covariate was not performed due to the small sample size and convergence issues.

| **Dataset** | **Land use**  **category** | **Time**  **variable** | **χ^2^_1_** | **P-value** |
| --- | --- | --- | --- | --- |
| night | forest | Standardized time | 0.03 | 0.86 |
|  | fields |  | 2.37 | 0.12 |
|  | plantations |  | 0.42 | 0.52 |
|  | village |  | 0.82 | 0.37 |
|  | forest | Total hours | 0.28 | 0.60 |
|  | fields |  | 0.77 | 0.38 |
|  | plantations |  | 0.00 | 0.97 |
|  | village |  | 0.04 | 0.85 |
| slow | forest | Standardized time | 0.52 | 0.47 |
|  | fields |  | 2.92 | 0.09 |
|  | plantations |  | 0.33 | 0.57 |
|  | village |  | 0.91 | 0.34 |
|  | forest | Total hours | 0.72 | 0.40 |
|  | fields |  | 1.06 | 0.30 |
|  | plantations |  | 0.00 | 0.95 |
|  | village |  | 0.07 | 0.79 |

Table S9: Anti-*Anopheles* saliva antibodies univariate models results and their significance value (N = 197). Variables with significant P-value (P < 0.05) are indicated in bold.

| Qualitative | Seroprevalence (%) | CI | χ^2^ | df | P-value |
| --- | --- | --- | --- | --- | --- |
| Season |  |  | 35.36 | 1 | **< 0.0001** |
| *dry* | 30.5 | [23.2 -38.5 %] |  |  |  |
| *rainy* | 66.4 | [57.2 - 74.8%] |  |  |  |
| Age category |  |  | 0.37 | 2 | 0.83 |
| *younger [≤ 20 years old]* | 43.9 | [33.0 - 55.3%] |  |  |  |
| *mid [21-39 years old]* | 48.2 | [38.8 - 57.8%] |  |  |  |
| *older [≥ 40 years old]* | 45.9 | [34.3 - 57.9%] |  |  |  |
| Village category |  |  | 3.41 | 2 | 0.18 |
| *inside forest* | 57.8 | [42.2 - 72.3%] |  |  |  |
| *forest fringe* | 40.7 | [30.0 - 52.2%] |  |  |  |
| *outside forest* | 45.8 | [37.5 - 54.3%] |  |  |  |
| Malaria status |  |  | 1.76 | 1 | 0.19 |
| *positive* | 50.4 | [41.6 - 59.2%] |  |  |  |
| *negative* | 42.3 | [33.9 - 51.1%] |  |  |  |
| ST in forest slow | - | - | 1.45 | 1 | 0.23 |
| ST in forest at night | - | - | 1.71 | 1 | 0.19 |
| ST in fields slow | - | - | 2.05 | 1 | 0.15 |
| ST in fields at night | - | - | 1.82 | 1 | 0.18 |
| ST in plantations slow | - | - | 0.21 | 1 | 0.64 |
| ST in plantations at night | - | - | 0.14 | 1 | 0.70 |
| ST in village slow | - | - | 2.49 | 1 | 0.11 |
| ST in village at night | - | - | 2.60 | 1 | 0.11 |
| Quantitative | **Average ΔOD** | **SD** | **χ^2^** | **df** | **P-value** |
| Season |  |  | 58.48 | 1 | **< 0.0001** |
| *dry* | 0.48 | 0.20 |  |  |  |
| *rainy* | 0.98 | 0.61 |  |  |  |
| Age category |  |  | 0.75 | 2 | 0.68 |
| *younger [≤ 20 years old]* | 0.66 | 0.47 |  |  |  |
| *mid [21-39 years old]* | 0.72 | 0.53 |  |  |  |
| *older [≥ 40 years old]* | 0.69 | 0.49 |  |  |  |
| Village category |  |  | 5.55 | 2 | 0.06 |
| *inside forest* | 0.87 | 0.58 |  |  |  |
| *forest fringe* | 0.62 | 0.42 |  |  |  |
| *outside forest* | 0.69 | 0.50 |  |  |  |
| Malaria status |  |  | 0.3 | 1 | 0.61 |
| *positive* | 0.72 | 0.52 |  |  |  |
| *negative* | 0.68 | 0.48 |  |  |  |
| ST in forest slow | - | - | 1.35 | 1 | 0.24 |
| ST in forest at night | - | - | 1.65 | 1 | 0.20 |
| ST in fields slow | - | - | 2.99 | 1 | 0.08 |
| ST in fields at night | - | - | 2.57 | 1 | 0.11 |
| ST in plantations slow | - | - | 0.82 | 1 | 0.36 |
| ST in plantations at night | - | - | 0.74 | 1 | 0.39 |
| ST in village slow | - | - | 2.58 | 1 | 0.11 |
| ST in village at night | - | - | 2.43 | 1 | 0.12 |

Table S10: Average ΔOD per village (N = 270).

| Village | Village category | N | Average ΔOD |
| --- | --- | --- | --- |
| Gaty | Inside forest | 10 | 1.09 |
| Ohchra |  | 8 | 0.42 |
| Ohtrone |  | 1 | 0.43 |
| Sraelvy |  | 26 | 0.94 |
| Poucha | Forest fringe | 22 | 0.45 |
| Sraeampilkroam |  | 24 | 0.42 |
| Sraeampilleu |  | 35 | 0.86 |
| Sraektum | Outside forest | 128 | 0.68 |
| Sraepreas |  | 16 | 0.74 |
| *Total* | ***-*** | ***270*** | ***0.70*** |


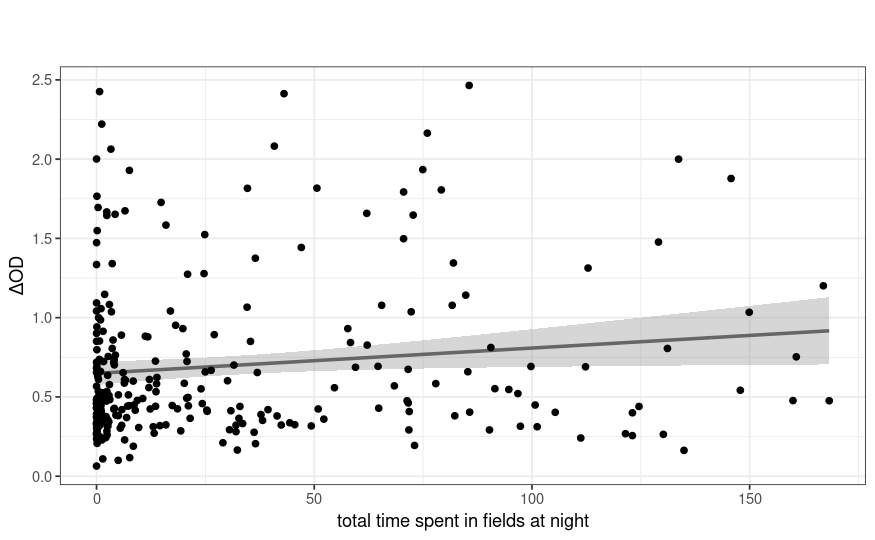


Figure S4: Association between ΔOD and the total time spent (in decimal hours) in fields at night by participants (N = 270).


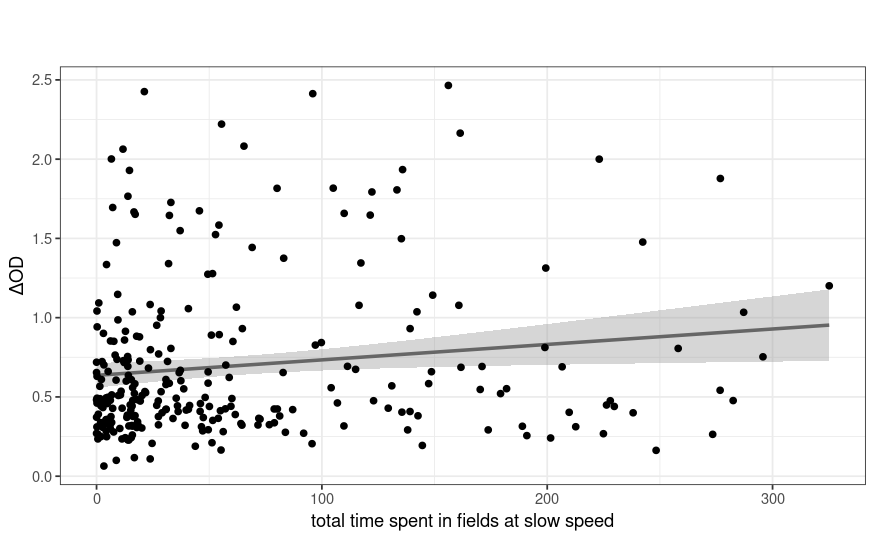


Figure S5: Association between ΔOD and the total time spent in fields (in decimal hours) at slow speed by participants (N = 270).

Table S11: GLMMs comparisons of serostatus and time spent in a category of land use with village of residence as a covariate. Time variable was extracted from optimal data, as night data (18:00 to 5:59) or slow data (<5 km/h) for all participants with a valid optimal GPS dataset (N = 270). Significant P-values are indicated in bold.

| **Dataset** | **Land use**  **category** | **Time**  **variable** | **χ^2^_1_** | **P-value** |
| --- | --- | --- | --- | --- |
| night | forest | Standardised time | 0.30 | 0.58 |
|  | fields |  | 2.25 | 0.13 |
|  | plantations |  | 2.71 | 0.10 |
|  | village |  | 0.54 | 0.46 |
|  | forest | Total hours | 0.50 | 0.48 |
|  | fields |  | 4.23 | **0.04** |
|  | plantations |  | 2.49 | 0.11 |
|  | village |  | 0.54 | 0.46 |
| slow | forest | Standardised time | 0.26 | 0.61 |
|  | fields |  | 2.23 | 0.14 |
|  | plantations |  | 2.95 | 0.09 |
|  | village |  | 0.48 | 0.49 |
|  | forest | Total hours | 0.83 | 0.36 |
|  | fields |  | 4.03 | **0.04** |
|  | plantations |  | 2.05 | 0.15 |
|  | village |  | 0.17 | 0.68 |

Table S12: Comparison of participants’ responses to questionnaire to their GPS data, for all land use categories (N = 197). Underlined numbers correspond to count of participants with discordant data between GPS and questionnaire.

|  | **Data from questionnaire** | |  |
| --- | --- | --- | --- |
| **Dataset from GPS device** | **visiting** | **not visiting** |  |
| visiting | 133 | 6 | forest |
| not visiting | 47 | 11 |  |
| visiting | 92 | 29 | plantations |
| not visiting | 48 | 28 |  |
| visiting | 95 | 94 | fields |
| not visiting | 1 | 7 |  |
